# Intrinsic neural activity predisposes susceptibility to a body illusion

**DOI:** 10.1093/texcom/tgac012

**Published:** 2022-03-12

**Authors:** Tzu-Yu Hsu, Ji-Fan Zhou, Su-Ling Yeh, Georg Northoff, Timothy Joseph Lane

**Affiliations:** Graduate Institute of Mind, Brain, and Consciousness, Taipei Medical University, 12th Floor, No. 172-1, Sec. 2, Keelung Rd., Taipei, Taiwan; Brain and Consciousness Research Centre, TMU Shuang Ho Hospital, llth Floor, Office 1105, Second Medical Building, No.291, Zhongzheng Rd., Zhonghe District, New Taipei City, 23561, Taiwan; Department of Psychology and Behavioral Sciences, Zhejiang University, Xiwu Teaching Building, Xixi Campus, Zhejiang University, 148 Tianmushan Road, Hangzhou, China; Department of Psychology, National Taiwan University, No. 1, Sec. 4, Roosevelt Rd., Taipei 10617, Taiwan; Graduate Institute of Brain and Mind Sciences, National Taiwan University, College of Medicine, No.1, Sec.1, Ren-Ai Rd., 15th Floor, Rm 1539, Taipei, Taiwan, Taipei 10051, Taiwan; Neurobiology and Cognitive Science Center, National Taiwan University, Taipei, Taiwan; Center for Artificial Intelligence and Advanced Robotics, National Taiwan University, Room 524, EE Building II, No. 1, Sec. 4, Roosevelt Ed., Taipei 10617, Taiwan; Institute of Mental Health Research, University of Ottawa, 1145 Carling Avenue, Ottawa, ON, Canada; Graduate Institute of Mind, Brain, and Consciousness, Taipei Medical University, 12th Floor, No. 172-1, Sec. 2, Keelung Rd., Taipei, Taiwan; Brain and Consciousness Research Centre, TMU Shuang Ho Hospital, llth Floor, Office 1105, Second Medical Building, No.291, Zhongzheng Rd., Zhonghe District, New Taipei City, 23561, Taiwan; Institute of European and American Studies, Academia Sinica, No. 128, Sec. 2, Academia Rd., Nankang, Taipei 115, Taiwan

**Keywords:** EEG, intrinsic neural activity, rubber hand illusion, self-relatedness, α band power

## Abstract

Susceptibility to the rubber hand illusion (RHI) varies. To date, however, there is no consensus explanation of this variability. Previous studies, focused on the role of multisensory integration, have searched for neural correlates of the illusion. But those studies have failed to identify a sufficient set of functionally specific neural correlates. Because some evidence suggests that frontal α power is one means of tracking neural instantiations of self, we hypothesized that the higher the frontal α power during the eyes-closed resting state, the more stable the self. As a corollary, we infer that the more stable the self, the less susceptible are participants to a blurring of boundaries—to feeling that the rubber hand belongs to them. Indeed, we found that frontal α amplitude oscillations negatively correlate with susceptibility. Moreover, since lower frequencies often modulate higher frequencies, we explored the possibility that this might be the case for the RHI. Indeed, some evidence suggests that high frontal α power observed in low-RHI participants is modulated by δ frequency oscillations. We conclude that while neural correlates of multisensory integration might be necessary for the RHI, sufficient explanation involves variable intrinsic neural activity that modulates how the brain responds to incompatible sensory stimuli.

## Introduction

The body is malleable, albeit more for some than for others. Even lifeless objects can be experienced as belonging to self. Tastevin (1937) suggested that ersatz hands can be experienced as belonging to self when participants simply look at them. Then under experimental conditions, Botvinick and Cohen (1998) formalized and replicated induction of this “rubber hand” illusion (RHI). Subsequently, the RHI has been extensively replicated, by various techniques, multiple times, and for many purposes (Makin et al. 2008; Ehrsson 2009, 2012; Tsakiris 2010; deVignemont 2017; Braun et al. 2018; Riemer et al. 2019). But we do not yet know what intrinsic neural activity makes possible this illusion.

On the standard version of the RHI, an artificial hand is experienced as belonging to self when subjects see it being stroked, concurrent with feeling strokes applied to the occluded, real hand. But when visual and tactile sensations are asynchronous—the common control condition—the illusion either fails to occur or is less vivid (Tsakiris 2010; Riemer et al. 2019; cf. Lush et al. 2021; Lush and Seth 2022; Ehrsson et al. 2022). The RHI paradigm has become a cornerstone of investigations into the science of self, and the distinctive ownership illusion tends to be explained as the result of multisensory integration of a type whereby vision prevails over other senses (Ehrsson 2012; Tsakiris 2017; Braun et al. 2018).

Subjective report evidence is adduced from questionnaires that generate data amenable to psychometric analyses (e.g. Longo et al. 2008; Romano et al. 2021). Behavioral or physiological evidence is derived from measures like proprioceptive drift, skin conductance, time of onset or duration, and temperature (e.g. Ehrsson et al. 2004; deHaan et al. 2017; Lane et al. 2017; Yeh et al. 2017; Critchley et al. 2021). The multisensory integration hypothesis suggests that neural activity that mediates the experience seems to involve the premotor cortex, the intraparietal sulcus, the anterior insula, and the sensorimotor cortex (Ehrsson et al. 2004; Kammers et al. 2009; Petkova et al. 2011; Bekrater-Bodmann et al. 2014; della Gatta et al. 2016; Limanowski and Blankenburg 2016; Lira et al. 2018; Peviani et al. 2018). It has not yet been possible, however, to identify a sufficient set of functionally specific neural correlates; in fact, even when employing the higher time resolution of EEG, results have been inconsistent (for a summary of the relevant studies, see Rao and Kayser 2017).

Because neural correlates of consciousness (NCCs) have failed to explain variable susceptibility to the RHI, we conjecture that sufficient explanation will require inclusion of intrinsic neural activity. Moreover, because investigations of intrinsic activity have proven useful in identifying neural instantiations of self-relatedness (Gusnard et al. 2001; Smallwood and Schooler 2015; Qin et al. 2016), we focus on the self-relatedness feature of the RHI, the feeling that the ersatz hand belongs to self (Lane 2015). In short, we hypothesize that self-relatedness features of the brain’s resting state activity can help explain observed variability.

In other words, to account for interpersonal variability, we propose that the explanatory framework must be expanded beyond NCCs and include neural predispositions. Indeed, among the more prominent types of neural activity that help track instantiation of self is resting-state α (alpha) power, RSAP (Kraus et al. 2021). The resting state’s—the brain’s spontaneous, intrinsic activity—frontal α power can be used to predict whether subjects will identify external objects as self-related (Bai et al. 2016; Lane et al. 2016). In this instance, the external object just is the ersatz hand.

Other investigators of body illusions have called attention to the significant role played by α band power, especially when it involves regions that contribute to self-related processing, like the medial prefrontal cortex (Lenggenhager et al. 2011). Indeed, studies targeting diverse phenomena have suggested that self-related processing involves intrinsic frontal activity (Lehmann et al. 2001; Ben-Simon et al. 2008; Fingelkurts et al. 2012; Knyazev et al. 2012; Knyazev 2013; Lane 2020). What we propose is that to the degree that self-related neural processing tracks the self, this neural processing might be able to serve as a proxy for assessing stability of the subjective sense of one’s own body. That is to say, the more stable the self, the less malleable it is, even in the face of mismatched, synchronous stimuli encountered in body illusion paradigms.

Clearly, α power can be employed to track numerous phenomena (e.g. Thut et al. 2006; Hanslmayr et al. 2007; Romei et al. 2008; Samaha and Postle 2015; Piantoni et al. 2017; Clayton et al. 2018; Hutchinson et al. 2021). More generally, α rhythms can play an inhibitory role that includes modulation of temporal windows, potentially constraining multisensory integration of the type conjectured to play a role in the RHI (Klimesch et al. 2007; Cecere et al. 2015; Bastiaansen et al. 2020). So our claim is not that α represents or is the neural realization of self. Instead, we treat it as one proxy whereby self-related neural activity can be tracked (Lane 2020). Specifically, our “hypothesis” is that frontal RSAP can be used to track the degree to which a subject’s boundaries between self and an inanimate object (viz., the rubber hand) are susceptible to blurring. To cast this idea in colloquial terms: if we take frontal RSAP as a means of tracking, the persistence of self-boundaries, despite the use of multisensory stimuli designed to trick the brain’s routine integrative tendencies, the greater the RSAP, the less likely a brain is to succumb to the illusion. In a word, the greater the frontal RSAP, the less likely a participant is to feel the rubber hand belongs to self.

Finally, lower frequencies often modulate higher frequencies (Buzsáki and Draguhn 2004; Canolty et al. 2006; Brookes et al. 2011), enabling integration of information from distal brain regions at low energy costs (Freeman and Rogers 2002; Vanhatalo et al. 2004; Buzsáki 2019). Moreover, intrinsic temporal properties, like the “nesting” of higher within lower frequencies, might contribute to our sense of self (Kolvoort et al. 2020): in part, self may be realized in virtue of intrinsic neural integration between higher and lower frequencies (Wolff et al. 2019; Wolff et al. 2022). And for psychotic states wherein boundaries between self and object are blurred (Lane 2014), patient RHI responses differ from those of healthy controls (Peled et al. 2000; Thakkar et al. 2011; Germine et al. 2013), with the patients evincing changes in low frequency power during the eyes-closed (EC) resting state (Ranlund et al. 2014; Newson and Thiagarajan 2019). Accordingly, we explored the slower frequency oscillations δ and θ, along with the possibility that RSAP might be modulated by the phase of either δ or θ.

In sum, we investigated EEG data, examining RSAP and its possible relatedness to RHI susceptibility, in particular the ownership feature. In addition, we explored the possibility that RSAP might be modulated by lower frequencies: toward that end, we investigated phase–amplitude coupling (PAC) for δ and θ with α frequency oscillations. Therefore, our principal hypothesis was that EC RSAP, both before and after performance of the RHI task, would evince a negative correlation with participant reports that the ersatz hand belongs to self. As an adjunct, we explored the slower frequency oscillations as well as PAC, to determine whether it might also help explain susceptibility to the illusion.

## Materials and methods

### Participants

Twenty-four right-handed college students from the National Taiwan University community participated in this study. In order to estimate the required sample size, a post hoc power analysis was performed (Hulley et al. 2013). Following Kanayama et al. (2017), in order to ensure sufficient power for identifying a correlation between neural activity and the RHI, 24 participants are required (α = 0.05, power = 0.90, *r* = 0.61). In order to test our main hypothesis, which involves distinguishing between high- and low-RHI groups, we recruited 24 subjects, 12 for each group. All had normal or corrected-to-normal vision and all were neurologically unimpaired. This study was approved by the Research Ethics Committee, National Taiwan University. All participants gave informed consent.

### Experimental procedures

This study comprised 2 stages: In stage 1, the standard RHI induction task is employed (see below). For this stage, participants indicated illusion onset by pressing their feet to pedals, in a manner that we used in our prior investigations (Lane et al. 2017; Yeh et al. 2017). In stage 2, EEG experimental procedures are employed (see below). The purpose of dividing the experiment into 2 stages was to first identify distinct subsets of participants—high and low susceptibility. Marking this distinction was in preparation for the second stage, in which the main hypothesis—viz. high and low would evince intrinsic neural activity differences—would be tested.

### Stage 1: the standard RHI induction task

Participants were tested, individually, in a small, quiet room. The experimenter sat in front of the participant, who was seated with both hands placed on a tabletop. Participants were asked to insert their hands into a black cardboard tube, so that they would be hidden from view. A towel was placed over the tube and in such a way that it would conceal participants’ elbows and forearms, as well as the space separating the rubber hand from the body (see Lane et al. 2017, Fig. 1). Then, the experimenter proceeded to use 2 paintbrushes to stroke corresponding fingers of rubber and biological hands, at an approximate rate of 1 per 2 s. Because evidence suggests right hemispheric dominance for the experience of body ownership (Ocklenburg et al. 2010), strokes were applied to the real left hand and a corresponding left, rubber hand. The procedure ceased either at 15 s after illusion onset or after 3 min, for those participants who did not experience the illusion. It was in this way that we identified the distinct subsets of susceptibility, in preparation for stage 2.

**Fig. 1:**
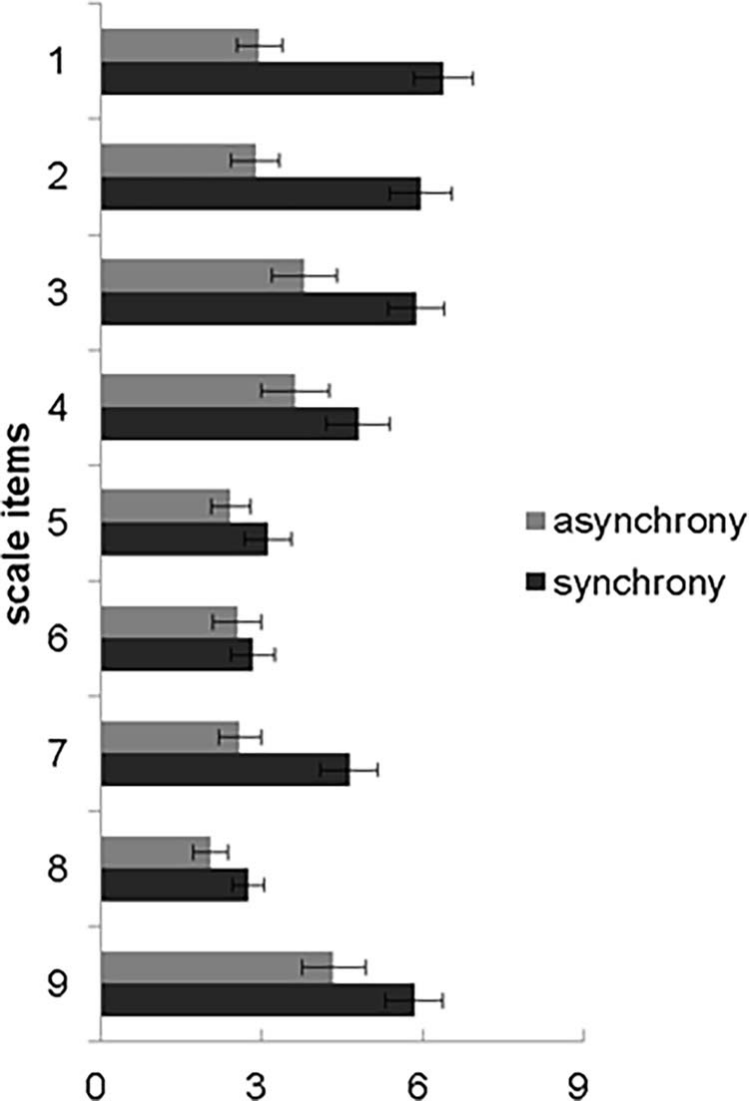
Questionnaire scores for synchronous and asynchronous conditions. Items #1–#3 were probed in order to investigate the ownership experience, with special attention given to #3, which explicitly concerns ownership.

Two conditions were administered for each participant: synchronous and asynchronous stroking, with the order counterbalanced across participants, and the asynchronous condition was treated as the control. In both conditions, the experimenter stroked the rubber hand and participants’ real hands, attempting to induce the RHI. Participants were required to keep looking at the rubber hand and avoid postural adjustments. After stroking of the hands was completed, participants were asked to fill out questionnaires.

The main questionnaire used for this experiment was adapted from Botvinick and Cohen (1998). It was used to evaluate whether, or the degree to which, the illusion was experienced in the synchronous condition, relative to the asynchronous condition. Responses for each item were indicated on a 10-point scale. Questionnaire scores for each participant were assessed by subtracting synchronous from asynchronous scores. Because in this stage we used the standard control condition for assessing susceptibility, it was this questionnaire data that we matched to the EEG data collected in stage 2.

### Stage 2: EEG experimental procedure

All participants underwent the RHI task procedure; each time the stroking continued for 1 min. The duration of stroking was 1 min, both because of our findings from stage 1 and because other investigations of the RHI have found that it can occur in less than 12 s (Ehrsson et al. 2004; Tsakiris and Haggard 2005; Arzy et al. 2006; Tsakiris et al. 2007). Moreover, because the standard RHI induction task in stage 1 enabled us to distinguish between 2 groups—those who are susceptible and those who are not—we did not use the standard control condition: that is, only synchronous stroking was employed, 5 times for each participant.

As for EEG measurements of intrinsic neural activity, a total of 1 min of eyes-open (EO) and 1 min of EC resting activity were measured, both before and after the induction of RHI. Both EO and EC were sustained for 20 s and repeated 3 times: in each instance, we began with EC and then alternated between EC and EO (viz. EC, EO, EC, EO, EC, EO) 3 times. During the EC, participants were instructed to close their eyes and relax without thinking of anything in particular; whereas, during EO, participants were required to fixate on a cross. Importantly, 1 min of recording is sufficient to detect the low frequency, intrinsic oscillations, which are the target of our investigation (Lu et al. 2007; Mantini et al. 2007; Karamacoska et al. 2017).

### E‌EG recording and analysis

EEG activity was recorded with Ag/AgCl electrodes mounted in an elastic cap (Electrocap International) using a 64-electrode arrangement, following the International 10-20 System. Two additional electrodes were referenced to the left and right mastoid. Vertical eye movements were recorded from electrodes above and below the right eye and horizontal electrooculograms were also recorded from electrodes at the outer canthi. Electrode impedances were kept below 10 kΩ for all electrodes, and amplifier bandpass was 0.1–100 Hz. Data were recorded with Neuroscan 4.2 software, with a sampling rate of 1,000 Hz.

All data analysis was performed off-line using EEGLAB (Delorme and Makeig 2004) and custom MATLAB (MathWorks) scripts. The continuous EEG was then segmented into 20-s long epochs. Independent component analysis was applied to remove eye-movement-induced artifacts (blinks or saccades). Also, epochs containing excessive noise or drift (±100 μV) at any electrode were excluded. The power of the signals from each channel was computed in 1 and 40 Hz (Roach and Mathalon 2008) using Morlet wavelet convolution with 3 cycles and 0.2 Hz step. Finally, power was calculated by converting the signal to a decibel (dB) scale, by multiplying logarithm (10^*^log 10[power]). All contrast between conditions was tested with nonparametric permutation testing; this corresponds to a cluster-level threshold of *P* < 0.01.

In order to explore cross-frequency coupling during intrinsic activity, i.e. PAC between the pair of frequencies (Canolty et al. 2006), we adopted a modified version of the modulation index (PACz, Cohen 2014). This was done in order to control for potential confounds and render the data amenable to statistical evaluation (Cohen 2014). First, the raw signal was separated into α power and δ phase through zero-phase band-pass filtered from 8 to 13 Hz and 1 to 3 Hz, respectively; the Morlet wavelet was then applied to estimate the amplitude of α frequency oscillation and the phase of δ range oscillation. Second, the modulation index (Canolty et al. 2006) was used to identify coupling between α amplitude and δ phase. The analysis was done with selected electrodes from anterior sites (AF3, AF4, F1, Fz, and F2); anterior sensors were selected in order to test our hypothesis concerning frontal α activity. Third, surrogate signals (*n* = 1,000) were computed to create randomly permuted power time series distribution of PAC values. We did not use the raw PAC values; instead, PACz was estimated through PAC value that was normalized with respect to randomly permuted PAC values. The normalized PAC—the PACz—was normalized by *z* values (Cohen 2014). Therefore, we adopt the *z*-score to identify PACz’s α threshold of *P* < 0.05. To control for type I errors when conducting multiple comparisons, the false discovery rate (FDR) method (Benjamini and Hochberg 1995; Groppe et al., 2011) was applied—a *P*-value of less than 0.05 survived FDR-controlling procedures.

## Results

### Synchrony/asynchrony manipulations for the RHI

To ensure that we have replicated the preponderance of previous RHI studies, for each item 2-way Greenhouse–Geisser adjusted, repeated-measures analysis of variance was applied, enabling comparison of questionnaire scores across synchronous and asynchronous conditions. Bayesian statistics are also provided to represent the strength of evidence for the null versus the alternate hypothesis. For our purposes, the null hypothesis is H0; scores for synchronous and asynchronous conditions do not differ. And, the alternate hypothesis is H1; scores for the synchronous and asynchronous conditions differ.

A large positive Bayes factor (BF_10_) provides a quantitative value in support of H1, whereas a small Bayes factor provides evidence for H0. For the questionnaire scores from Botvinick and Cohen (1998), a significant main effect of synchrony was found: *F*(1, 23) = 35.547, *P* < 0.001, $\eta $^2^ = 0.144; BF_10_ = 3.382 × 10^12^. Scores for the synchronous condition (*M* = 4.685, SD = 1.450) were higher than those for the asynchronous condition (*M* = 3.019, SD = 0.745; Fig. 1). The main effect of item was also significant: *F*(3.487, 80.208) = 12.16, *P* < 0.001, $\eta $^2^ = 0.192; BF 10 = 2.207 × 10 ^12^. In addition, a significant interaction between synchrony and item also was found: *F*(3.475, 79.914) = 8.143, *P* < 0.001, $\eta $^2^ = 0.054; BF_10_ = 4.650 × 10^3^. Excluding items 5 and 6 (*P*s > 0.05), post hoc analysis evinced that scores for items in the synchronous condition were significantly higher than scores for the asynchronous condition (*P* < 0.01). Hence, our findings were in line with the preponderance of previous RHI studies. Of most direct relevance to our investigation item 3, along with items 1 and 2, those that probe the sense of belonging or ownership also showed significant, synchrony-induced RHI. Although a recent concern about this methodology has incited some debate (Lush et al. 2021; Ehrsson et al. 2022; Lush and Seth 2022), we adopted subtracted scores (synchronous–asynchronous) to represent questionnaire scores for the analysis that follows. We address this debate in Section 4.

### Intrinsic neural activity before and after RHI induction, and EO/EC induced frequency oscillation differences

**Fig. 2:**
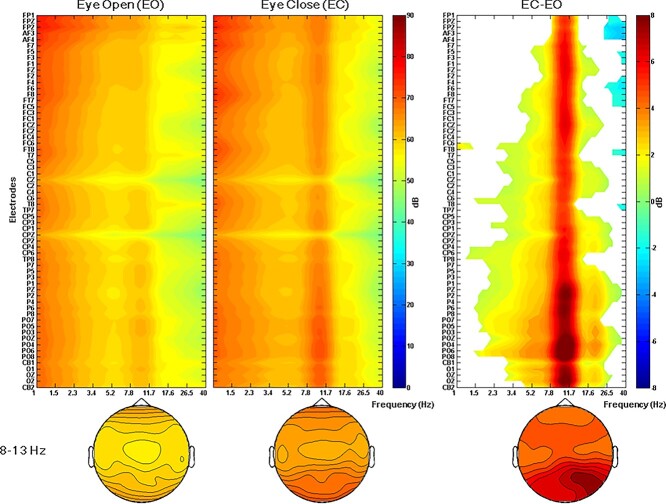
Ground averaged power spectrums during resting states. Power spectra were plotted as a function of electrodes and, from left to right, panels are depicted for: EO, EC, and the difference between EC and EO. Bottom panels depict topography of α frequency power for each condition. The α frequency power differences are stronger, progressively, from frontal to posterior electrodes.

Before examining the relationship between intrinsic neural activity and RHI susceptibility, we examined (a) whether intrinsic neural activity, before and after RHI induction, differs; and (b) whether alpha band oscillations, for EO and for EC, differ. Intrinsic neural activity for multiple conditions was transformed into time–frequency power. As for (a), we compared time–frequency power of intrinsic neural activity in EO and EC conditions, both before and after RHI induction. No significant difference for intrinsic neural activity, before and after RHI induction, was observed. Therefore, EO and EC intrinsic neural activity from before and after RHI induction were pooled, separately, for subsequent analysis. As for (b), time–frequency power across different electrodes, as a function of 1–40 Hz is shown in Fig. 2. The magnitude of α frequency power was significantly higher for EC, relative to EO conditions, across whole electrodes. Moreover, the right panel in Fig. 2 (permutation test, *P* < 0.01) shows that the magnitude of α frequency power across multiple electrode sites significantly increased during EC.

### The relationship between intrinsic neural activity and RHI

**Fig. 3:**
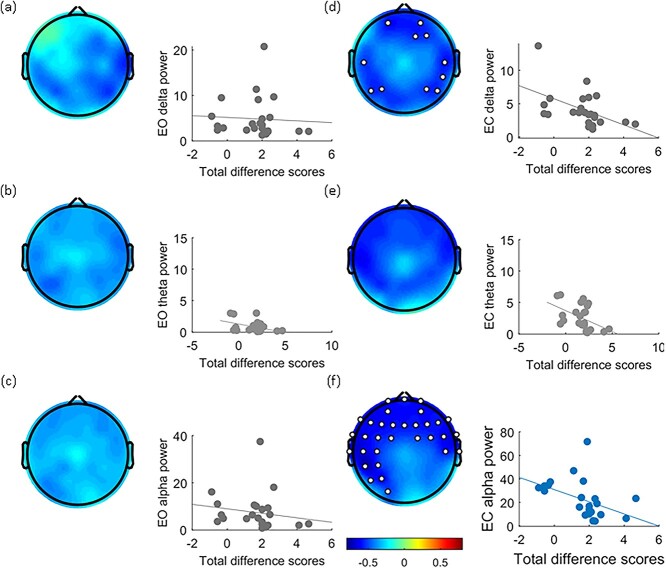
Correlation between total difference scores with EO/EC α power. The left panel is EO and the right panel is EC condition. All topography of the EEG showed rho values between the scores across electrodes and power across frequency bands. δ, θ, and α power are listed from top to bottom. Take note of (f): A significant negative correlation between total difference scores and α power was only evinced for frontal and LLP electrode sites (white dots, *q* < 0.05), in the EC condition. To depict the negative correlation between alpha power and the total difference score, we combined alpha power from those significant electrodes under the EC condition and correlated with the total difference score (f). As for (a) to (e), no significant correlation was observed. All electrode sites were averaged in order to calculate relationships to total difference scores.

We further examined whether the subjective experience of RHI correlates with the amplitude of α frequency power. Accordingly, we conducted a spearman’s rho correlation on the total difference scores and the amplitude of α frequency power across electrodes under both EO and EC conditions. Additionally, because self-relatedness has been conjectured to promote multisensory integration in virtue of integration among faster and slower frequencies (Wolff et al. 2019), the same analysis was applied to 2 slower frequency bands, δ and θ. Results evinced significant negative correlation between total difference scores and amplitude of α power, in frontal to left lateral parietal (LLP) electrodes in the EC condition (Fig. 3f, *q* < 0.05) but not in the EO condition (Fig. 3c). Intriguingly, some electrodes in the EC condition also evinced a significant negative correlation between total difference scores and amplitude of δ power (Fig. 3d), though here too not in the EO condition (Fig. 3a). θ power, however, did not evince any correlation with total difference scores (Fig. 3b and e).

### The relationship between α power and ownership illusion items

**Fig. 4:**
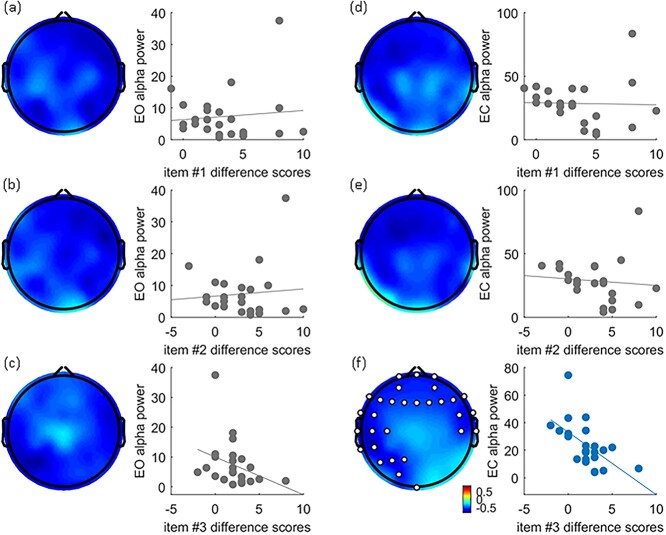
Correlation between item #1–3 difference scores with EO/EC α power. The left panel is EO and the right panel is EC condition. All topography of the EEG showed rho values between the difference scores for each item across electrodes and power across frequency bands. Items #1–#3 are listed from top to bottom. Take note of (f): A significant negative correlation between item #3 difference scores and α power was only evinced for frontal and LLP electrode sites (white dots, *q* < 0.05) in the EC condition. To depict the negative correlation between the α power and the item #3 difference score, we combined α power from those significant electrodes under the EC condition and correlated with the total difference score (f). As for (a) to (e), no significant correlation was observed. All electrode sites were averaged in order to calculate relationships to total difference scores.

Having established that a significant correlation between α power and the total difference scores is evinced, we proceeded to determine which item’s difference scores might correlate with α power. We further examined whether the subjective experience of RHI—in particular the ownership values for items #1 to #3—correlates with the amplitude of α frequency power. Results evinced significant negative correlations between ownership scores for item #3 and amplitude of α power in frontal to LLP electrodes in the EC condition (*q* < 0.05, FDR, in Fig. 4f). Significant correlations were not evinced, however, for the EO condition (Fig. 4c) nor for the other questionnaire items (Fig. 4a–e). To depict the relationship between the α frequency power and ownership scores in the EC condition, α frequency power for significant electrodes was averaged, revealing a correlation with ownership scores, rho(23) = −0.62, *P* < 0.01. In other words, for the EC condition, α frequency power decreased as ownership scores increased. As above, similar analyses were conducted for δ and θ power but neither evinced significant correlations (see Supplementary Figs. 1 and 2).

### Different RHI cohorts evinced different levels of low- and high-frequency oscillation coupling in the EC condition

**Fig. 5:**
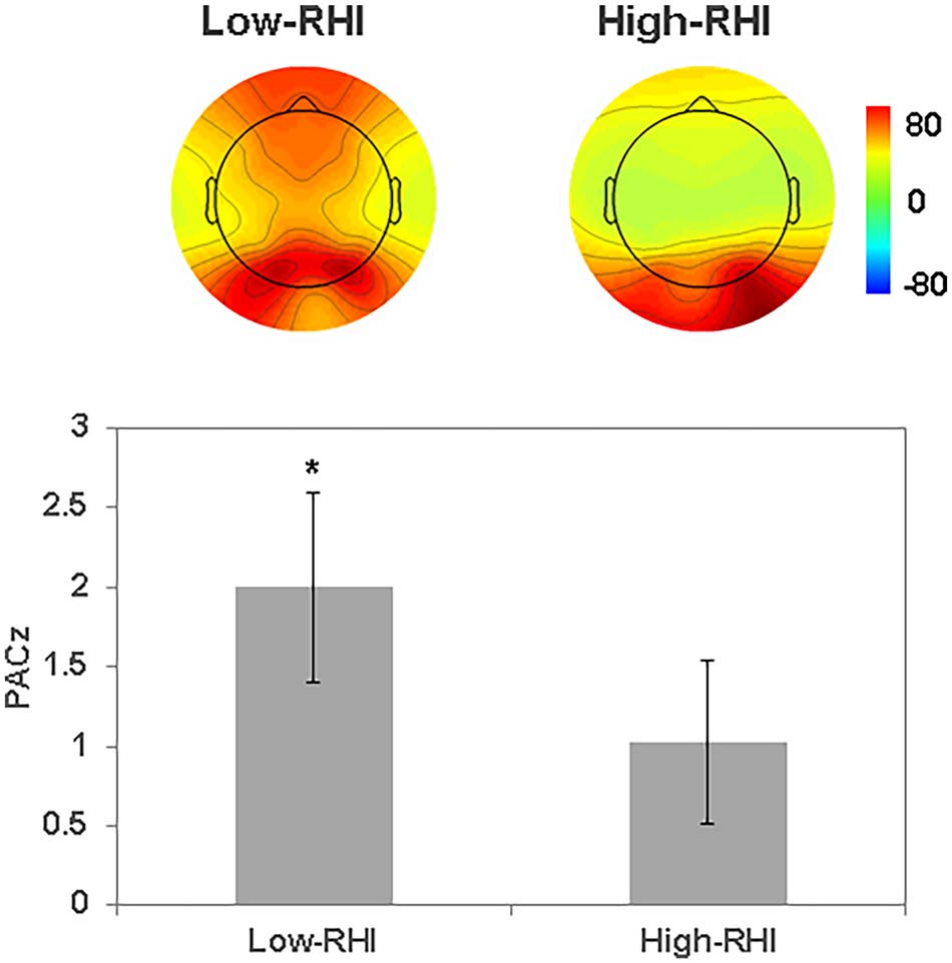
The δ phase and α amplitude coupling for low- and high-RHI performers in the EC condition. The top panel depicts topography of α frequency power in the EC condition. Left topography depicts low-RHI subjects; right topography, high-RHI subjects. Averaged PACz in low-RHI subjects was significantly larger than *Z*-score 1.96 (bottom left), whereas averaged PACz in high-RHI subjects was not (bottom right).

As an exploratory adjunct, we investigated the possibility of cross-frequency coupling between δ and α frequency oscillations that might correlate with the RHI. Accordingly, we calculated δ phase and α amplitude coupling (i.e. PACz) for the EC condition. Then, based on median splits of their ownership scores from item #3, participants were categorized into high- and low-RHI groups. Next we sought to determine whether cross-frequency coupling for intrinsic neural activity (viz. PACz) in the high-RHI or low-RHI groups differs from zero. Results showed that the PACz for the low-RHI group was significantly higher than zero (PACz = 1.986, *P* < 0.05), whereas PACz for the high-RHI group was not (PACz = 0.923, Fig. 5). These results suggest the possibility that for the EC condition and in the low-RHI group, communication across δ and α frequency oscillations is heightened.

## Discussion

To the best of our knowledge, this is the first study to describe an association between the brain’s intrinsic activity and the RHI susceptibility. Our findings suggest that neural predispositions—notably frontal EC RSAP—constrain susceptibility to the RHI. It appears to be the case that this intrinsic neural activity can be used to track the degree to which boundaries between self and an inanimate object are susceptible to blurring. Here we report data which suggest that the greater the frontal EC RSAP, the less likely a participant is to feel the rubber hand belongs to self.

More precisely, the magnitude of frontal α frequency power oscillations in the EC condition negatively correlates with the score of item #3 on the RHI questionnaire. That is the greater the α power, the less likely are subjects to affirm that “I felt as if the rubber hand was my own hand.” It seems to be the case that frontal, α power might carry information relevant to how malleable is the subjective sense of self-own body.

Furthermore, as an exploratory adjunct, we investigated the possibility that cross-frequency coupling might play a role in explaining variable susceptibility. In fact we did find some evidence to suggest that cross frequency communications differ between low- and high-RHI subjects. The high frontal α power observed in low-RHI participants appears to be modulated by δ frequency oscillations. This finding might imply that low-RHI participants are better able to preserve their original subjective sense of their bodies, because that sense is bolstered by communication among distal brain regions.

In a previous study we found that participants who evince less switch cost and higher attention-shift scores had faster RHI onset times; moreover, those who evince higher attention-shift scores experienced the RHI more vividly (Yeh et al. 2017). The findings suggest that a disposition for performing these executive functions contributes to illusion susceptibility. It follows then that if task switch and attention-shift are subserved by frontal neural activity, it might be that frontal α power inhibits them thereby inhibiting illusion susceptibility. If that finding is considered alongside of the explanation provided here—that EC RSAP makes the embodied-self more stable—it might be the case that neural substrates of these executive functions and of self-relatedness are working at cross-purposes.

Our intent is not to deny that NCCs for multisensory integration are relevant to the RHI. Our suggestion is that even more refined investigations of the RHI or other body illusions, if they focus on the task alone, are unlikely to provide a *sufficient* explanation of susceptibility. We submit that a necessary precondition might involve durability of the boundaries between self and the external, and one possible means of tracking this durability involves frontal EC RSAP. We further speculate that this activity might be nested in δ oscillations.

Neither is it our intent to deny that legitimate concerns have been raised about potential confounds to the RHI paradigm (Seth et al. 2021). Arguably, the most disconcerting, purported confound implicates demand characteristics, the ability of subjects to grasp and comply with intentions suggested by experimental design (Orne 1962; Marotta et al. 2016; Lush et al. 2020, 2021; Lush and Seth 2022). As this worry applies to the RHI, the concern is that participants who experience the rubber hand as belonging to self are exhibiting a capacity for phenomenological control: they respond to imaginative suggestions in such a manner that they confabulate the experiences implied by the experimental paradigm (Dienes et al. 2020).

Some evidence has been adduced to support the trait phenomenological control interpretation of the RHI (Lush et al. 2018, 2020, 2021), at least for the modest version of the claim: “RHI reports are, at least partially, likely to be driven by top-down phenomenological control” (Lush and Seth 2022). And there is reason to believe that this interpretation of the RHI investigations might help to explain some observed variability (Ehrsson et al. 2022). But we are not able to adjudicate between strong versions of the multisensory versus demand characteristic interpretations of the RHI. What our findings do suggest, however, is that in addition to multisensory integration, sufficient explanation of variable response involves susceptibility to suggestion of a specific type—blurring of the boundaries between self-own body and objects in the external world.

Finally, it could be argued that participant responses to some items on the questionnaire are a bit puzzling, for in the original study items 1–3 were taken to be illusion indicators (Botvinick and Cohen 1998). First, because our hypothesis concerned ownership and only item 3 refers explicitly to ownership, while items 1–2 concern touch referral (Reader et al. 2021), our analysis centered on item 3. Second, because the motivating hypothesis involved distinguishing between those who did and those who did not experience the illusion, for stage 2 we sought only to reconfirm that the distinction held; therefore, we did not collect additional subjective report data. Nevertheless, third, that items other than 1–3 evinced significant correlations remain puzzling. We interpret this result as implying that RHI phenomenology is more nuanced than can be adequately covered by the coarse-grained items that appear on standard questionnaires. Improved first person methodologies would stand to contribute much to research on embodiment (Lewis and Lloyd 2010; Moguillansky et al. 2013), in particular with regard to enhanced understanding of inter-individual difference in how the illusion is experienced (Carruthers 2019).

This work has several other limitations: First, although the sample size (*n* = 24) is in line with previous RHI studies (Tsakiris 2010) and equal to or greater than other RHI electrophysiological studies (Zeller et al. 2015; Guterstam et al. 2019; Sciortino and Kayser 2022), in the future it will be important to replicate these results in an independent sample. Second, EEG was not recorded during induction; therefore, we are not able to determine the relationship between intrinsic and task-performance neural activity. And, third, EC and EO conditions yielded different results; we speculate that this finding is due to variant constraints on temporal variability (Qin et al. 2018) or other temporal properties (Weng et al. 2020). But this too must await future confirmation.

The subjective experience of our bodies is malleable, albeit in varying degrees. The seeming inadequacy of online multisensory integration to explain inter-personal variation, as well as recent suggestions that trait variability might play an important explanatory role, suggests the importance of intrinsic neural activity. We hypothesized that frontal α amplitude can serve as a proxy for tracking the stability of self; as this applies to the RHI, EC RSAP can help assess susceptibility to a blurring of boundaries between self and external objects. Indeed, our findings suggest that variability in frontal α amplitude correlates with variable susceptibility to the RHI. An exploratory adjunct to our investigation also provides some evidence to suggest that frontal α amplitude is modulated by δ oscillations. Based on our findings, we submit that most instances of how this illusion is experienced require both an understanding of the NCCs of multi-sensory integration and an understanding of the intrinsic, neural predispositions that regulate the boundaries between self and the external world. If future studies apply this approach to full-body illusions, this intrinsic neural activity might also help to explain our sense of where and who we are.

## Supplementary Material

supp_Figure1_tgac012

supp_Figure2_tgac012

Supplementary_Figure_tgac012
