# Supplementary figures and images for: Intrinsic neural activity predisposes susceptibility to a body illusion
Source: Cereb Cortex Commun. 2022 Mar 12;3(1):tgac012. doi: 10.1093/texcom/tgac012 (PMC8976633; doi:10.1093/texcom/tgac012)

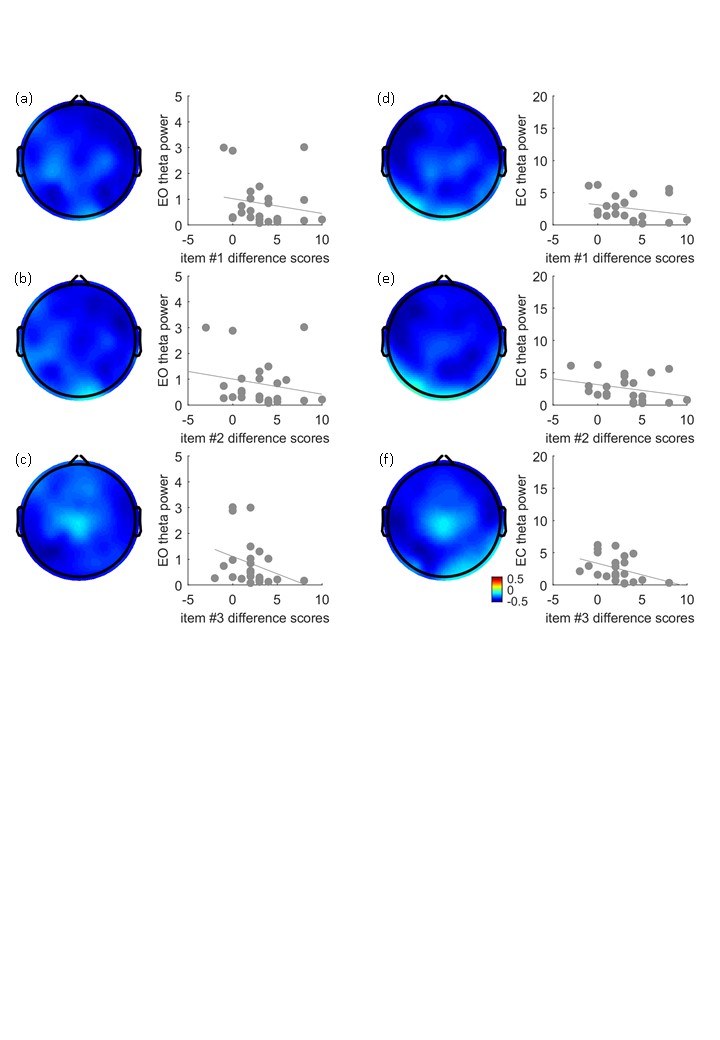

Supplement: supp_Figure1_tgac012 [file supp_figure1_tgac012.jpeg]

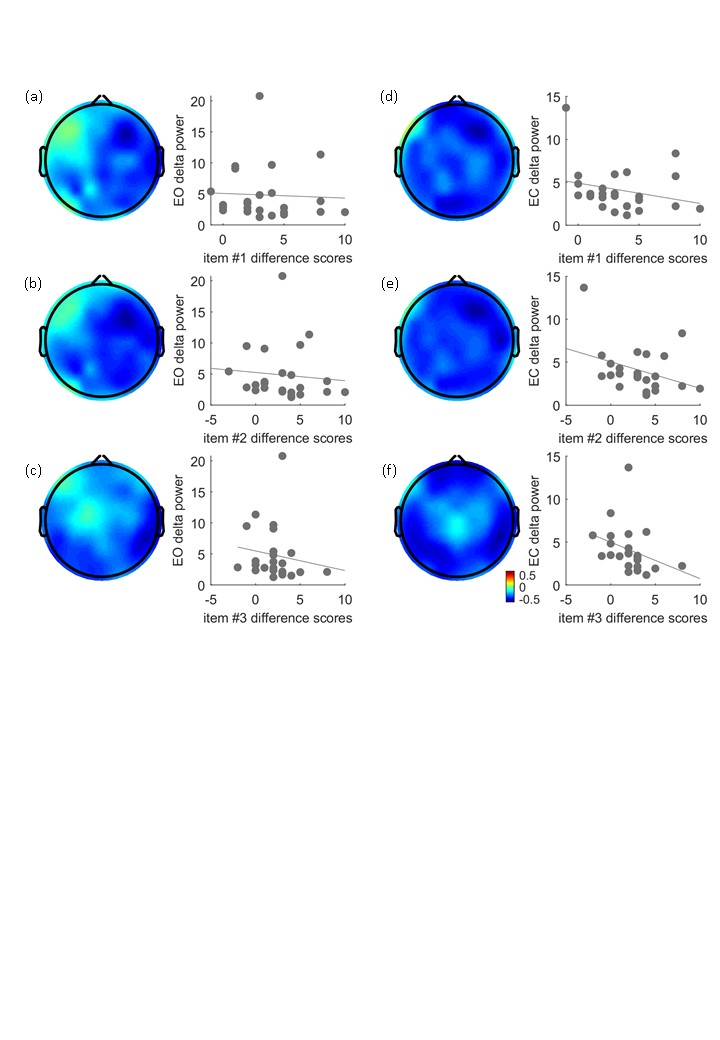

Supplement: supp_Figure2_tgac012 [file supp_figure2_tgac012.jpeg]
