# Supplementary material for: Intrinsic neural activity predisposes susceptibility to a body illusion
Source: Cereb Cortex Commun. 2022 Mar 12;3(1):tgac012. doi: 10.1093/texcom/tgac012 (PMC8976633; doi:10.1093/texcom/tgac012)
Supplement: Supplementary_Figure_tgac012 [file supplementary_figure_tgac012.docx]

Supplementary figure 1. Correlation between item #1-3 difference scores with EO/EC δ power.

Supplementary figure 2. Correlation between item #1-3 difference scores with EO/EC θ power.
